# Nicotine absorption during electronic cigarette use among regular users

**DOI:** 10.1371/journal.pone.0220300

**Published:** 2019-07-25

**Authors:** Jessica M. Yingst, Jonathan Foulds, Susan Veldheer, Shari Hrabovsky, Neil Trushin, Thomas T. Eissenberg, Jill Williams, John P. Richie, Travis T. Nichols, Stephen J. Wilson, Andrea L. Hobkirk

**Affiliations:** 1 Department of Public Health Sciences, Tobacco Center of Regulatory Science, Pennsylvania State University, College of Medicine, Hershey, PA, United States of America; 2 Department of Psychology, Center for the Study on Tobacco Products, Virginia Commonwealth University, Richmond, VA, United States of America; 3 Division of Addiction Psychiatry, Rutgers-Robert Wood Johnson Medical School, New Brunswick, NJ, United States of America; 4 Department of Psychology, Pennsylvania State University, University Park, PA, United States of America; Medical University of South Carolina, UNITED STATES

## Abstract

**Background:**

The capability of electronic cigarette devices (e-cigs) to deliver nicotine is key to their potential to replace combustible cigarettes. We compared nicotine delivery and subjective effects associated with the use of two classes of e-cigarettes and cigarettes.

**Methods:**

14 e-cigarette users were instructed to vape their own e-cigarette device every 20 seconds for 10 minutes while blood was drawn at 1, 2, 4, 6, 8, 10,12, and 15 minutes after initiating vaping. Users rated withdrawal symptoms and side effects before and after vaping. E-cigarette devices were classified as first-generation (same size as cigarette, no activation button) or advanced (larger than cigarette with an activation button). Separately, 10 cigarette smokers completed a similar protocol. Fisher’s Exact Test and two-sided t-tests were used as appropriate to determine differences in outcomes between first-generation e-cigarette users, advanced e-cigarette users, and smokers.

**Results:**

Compared to first-generation devices, advanced devices were associated with greater serum nicotine C_max_ (ng/ml) (11.5 v. 2.8, p = 0.0231) and greater nicotine boost (ng/ml) (10.8 v. 1.8, p = 0.0177). Overall, e-cigarettes users experienced a significant reduction in withdrawal and craving, although there were no significant differences between users of first-generation and advanced devices. Comparing e-cigarettes overall to cigarettes, cigarettes were associated with greater C_max_ (25.9 v. 9.0, p = 0.0043) and greater nicotine boost (21.0 v. 8.2, p = 0.0128).

**Conclusions:**

Advanced e-cigarettes delivered significantly more nicotine than first-generation devices but less than combustible cigarettes. Overall, e-cigarette use was associated with a reduction in withdrawal and craving with no reported side effects. The wide variation in nicotine absorption from different e-cigarette devices should be considered in studies of e-cigarettes for smoking cessation.

## Introduction

Tobacco use, particularly combustible cigarette smoking, remains the leading cause of premature death in the United States [1]. In 2009, the Family Smoking Prevention and Tobacco Control Act was implemented, allowing the United States Food and Drug Administration (FDA) to begin regulating tobacco products with the goal of protecting public health [2]. As the FDA works to design and implement regulations that will reduce the addictiveness and potential harms associated with combustible cigarette smoking, attention has shifted towards the role of a new tobacco product, generally referred to as an electronic cigarette (e-cigarette). It has been suggested that e-cigarettes could present an opportunity for smokers to quit combustible cigarettes [3] because e-cigarettes deliver nicotine in an aerosol that produces less toxicants than cigarette smoke [4]. The rate and amount of nicotine absorption from e-cigarettes is likely to influence both their ability to replace combustible cigarette use and their dependence potential.

Early e-cigarettes were classified into two types. E-cigarette devices similar in shape and size to a combustible cigarette were considered “Cigalikes”, or first-generation devices. Second-generation, or advanced devices, were those typically larger than first-generation devices and had more powerful batteries and a manual button to initiate heating of the coil prior to inhalation [5, 6]. Studies have reported that approximately a quarter of e-cigarette users are first-generation users while about three quarters of users use advanced devices, including modified devices [5, 7, 8].

Several studies have been conducted to measure the nicotine delivery of e-cigarettes. Pharmacokinetic studies of first-generation devices with e-cigarette naïve smokers have found first-generation nicotine exposure to be more similar to nicotine exposure from nicotine gum [9], a nicotinized inhalator [10], and even an unlit cigarette [11] than combustible cigarettes. In laboratory studies, the maximum plasma nicotine concentration levels with first-generation devices ranged from 1.3 to 5.36 ng/ml five to 24 minutes after use, depending on the nicotine concentration of the e-liquid [9–12]. Rates of nicotine delivery were slightly higher when the first-generation device was being used by experienced e-cigarette users compared with naïve users. For experienced e-cigarette users, average nicotine levels ranged from 4.87 to 6.77 ng/ml five to ten minutes after use [12–14]. Studies have also evaluated second generation or “advanced” devices. These studies evaluating advanced devices with experienced users found higher average plasma nicotine levels compared with first-generation devices that ranged from 6.59 to 17.9 ng/ml two to five minutes after use [13, 15–18].

In addition, the nicotine delivery of e-cigarettes has been compared to cigarettes. Some recent studies have found that the nicotine delivery of some advanced devices can meet or exceed the nicotine delivery of cigarettes [18, 19]. One of the most comprehensive studies of nicotine delivery from e-cigarettes to date in experienced e-cigarette users found newer e-cigarette products delivered nicotine more efficiently than cig-a-like brands, but none matched nicotine delivery from cigarettes [20].

Finally, experienced e-cigarette users have reported a significant reduction in urge to smoke and withdrawal symptoms after e-cigarette use [15, 16, 18, 21, 22] without significant increases in adverse effects like nausea and throat irritation [13, 15, 16, 18, 21, 22]. A comparison of first-generation and advanced devices found that use of advanced devices resulted in significantly fewer withdrawal symptoms and lower craving than first-generation devices at the end of a 65 minute ad-lib vaping session; however, there was no difference in withdrawal ratings after five minutes despite higher nicotine delivery with advanced devices [13]. Hiler et al. found that the nicotine concentration of the liquid significantly impacted withdrawal scores with those using the higher concentrations reporting the greatest reduction in withdrawal symptoms [18].

The current study aimed to provide further data on the nicotine delivery of e-cigarettes among experienced users using their own device. Specifically, we aimed to compare the nicotine delivery of varying e-cigarette devices, first-generation and advanced, with the nicotine delivery of cigarettes. In addition, we planned to evaluate the subjective effects associated with e-cigarette use between e-cigarette device types and cigarettes. This study is novel because it collected and measured blood nicotine levels at several time points while the participant was vaping, not just and pre and post vaping.

## Methods

### Current study

#### Participants

Electronic cigarette users were solicited via advertisements on e-cigarette websites and forums to complete an anonymous online survey about their e-cigarette use and preferences. Details of this survey have been published previously [5, 23]. Participants interested in volunteering for a laboratory study were invited to enter their contact details at the end of the survey. Participants who indicated interest were contacted via phone and screened for eligibility. Eligibility criteria for the lab portion of the study included being between the ages of 18 and 59 years old, using an e-cigarette for at least 30 days in their lifetime, using an e-cigarette for at least 20 of the last 28 days, and using an e-liquid nicotine concentration of at least 12 mg/ml. This nicotine concentration inclusion criterion was implemented to ensure that all participants would be using a device and liquid capable of delivering nicotine. Exclusionary criteria included experiencing a chronic condition (e.g, diabetes, hypertension, cancer) or cardiovascular or respiratory illness, current psychopathology or prescribed psychiatric medication, current drug or alcohol abuse, current pregnancy, or any difficulties donating blood in the past. From May 2014 to April 2015 eligible current e-cigarette users were invited to Penn State University, College of Medicine in Hershey, PA, or the Addiction, Smoking, and Health Lab at Penn State University in University Park, PA to complete the lab portion of the study using their own personal e-cigarette device. This study was approved by the Penn State University Institutional Review Board (IRB #40502). All participants completed a written consent procedure. Study data were collected and managed using REDCap electronic data capture tools hosted at the Penn State Milton S. Hershey Medical Center and College of Medicine. REDCap is a secure, web-based application designed to support data capture for research studies [24].

#### Study procedures

Participants deemed eligible via the phone screening were scheduled for a visit and asked to bring their personal e-cigarette and all necessary parts, including their preferred e-liquid. Participants were instructed to remain abstinent from traditional cigarettes for 4 days and from their e-cigarettes (or other nicotine containing products) and caffeine for 14 hours prior to the visit. Upon arriving at the lab, expired-air carbon monoxide (CO) was measured to verify no recent cigarette use (<8 ppm). Female participants of childbearing potential completed a pregnancy test prior to starting study procedures. Participants then completed a series of baseline questionnaires assessing demographic information, smoking history, and device characteristics, and completed both the 10-item Penn State Cigarette and Electronic Cigarette Index (PSCDI and PSEDCI) [23]. Scores on the PSCDI and PSECDI range from 0–20 (0–3 = not dependent, 4–8 = low dependence, 9–12 = medium dependence, 13+ = high dependence) [23]. In addition, participants completed computerized visual analog scale (VAS) questions regarding their anticipation of using their device, and any present physical symptoms, (e.g., nausea, dizziness, and dry mouth). The questions stated, “Please respond to each word or phrase with how you feel RIGHT NOW”) by clicking on the horizontal line ranging from “0-Not at all” to “100-Very much” for each item. Withdrawal related items were nervous, anxious, difficulty concentrating, restless, hunger, impatient, and feeling depressed. These items are valid DSM-5 criteria for measuring nicotine withdrawal and are also items included on the widely-used Minnesota Nicotine Withdrawal Scale [25]. Nicotine side effect related items were nauseous, dizziness, light-headed, sweaty, headache, and heart-pounding. An average score for withdrawal and nicotine side effects was created by averaging all items in each category.

After the baseline questionnaires were completed, trained nursing staff from the Penn State Clinical Research Center inserted a catheter into the participant’s arm to facilitate frequent blood draws. A 7ml blood sample was taken at baseline. Participants then began a vaping protocol in which they were instructed by a computer to take one puff on their e-cigarette every 20 seconds for 10 minutes totaling 30 puffs. The duration and volume of each puff was determined by the participant. This intensive standardized puffing protocol was utilized to highlight potential differences in nicotine delivery between varying device types given an equal number and timing of puffs. Blood was drawn while vaping at 1, 2, 4, 6, 8, and 10 minutes, and then at 12 and 15 minutes (2 and 5 minutes after completing the puffing session). Nursing staff removed the catheter at the completion of the vaping procedures and the participant was instructed to complete additional VAS questionnaires about their satisfaction with using their e-cigarette and any present physical symptoms.

The blood samples were processed at the completion of the visit and blood serum was frozen at -80°C. Liquid chromatography mass spectrometry was used to determine serum levels of nicotine. Nicotine was analyzed using a Phenomenex Synergi Polar RP column, 4.6 x 150 mm. Solvent A was 5mM ammonium acetate with 0.1% acetic acid added. Solvent B was 5mM ammonium acetate in methanol with 0.1% acetic acid added. The initial solvent composition of 80% solvent A/20% solvent B was held for 0.1 minute. A gradient was then run to 100% solvent B in 6.5 min. Nicotine eluted at approximately 4 minutes. The column was washed with 100% solvent B for 3.5 minutes before equilibrating at initial conditions. Nicotine was quantified using positive ion electrospray, monitoring the transition from m/e 163 → 130. The transition for the internal standard (d4 nicotine) was m/e 167 → 134. The temperature was 550⁰ C and the ionspray voltage was 1800 V. The limit of quantitation was 200pg/ml.

Magnetic resonance imaging was also completed by 11 of the 14 participants before and after the vaping procedures. The results of the MRI study were published previously [26, 27].

### Comparison study procedures

The blood nicotine levels collected during the current study were compared to the results from a previous study of combustible cigarette pharmacokinetics [28]. In the comparison study, measures of serum nicotine were collected from 10 smokers (without a psychiatric diagnosis) while they smoked one cigarette in a laboratory setting at the University of Medicine and Dentistry of New Jersey. In brief, participants were instructed to refrain from smoking cigarettes at 8:00pm the night before their scheduled lab session, resulting in at least 12 hours of abstinence prior to the session. Participants provided an exhaled CO measurement upon arrival to the session to confirm overnight abstinence (<15ppm). A venous catheter was inserted into the participant’s arm and a baseline blood sample was obtained. Participants smoked one of their own cigarettes ad libitum and the time spent smoking was measured from the first to the last puff. Blood samples were obtained at 1, 2, 4, 6, 8, 10, 20, 30, 60, 90 and 120 minutes after the first puff. Blood serum was frozen at -20°c and serum nicotine was quantified using a liquid chromatography-mass spectrometer. To ensure consistency across measurement techniques of the two labs (from the current study and the comparison study), 40 blood samples were analyzed separately in each lab and we found very good inter-lab agreement for nicotine (r = 0.92).

The full details of this study can be found in Williams et al [28].

### Data analysis

All participants were classified for analysis by the tobacco product used; e-cigarettes or cigarettes. E-cigarette users were further classified by their e-cigarette device type. E-cigarette devices larger than a traditional cigarette with a button to press prior to inhalation were considered advanced devices while those e-cigarette devices the same size or smaller than a traditional cigarette without a button were considered first-generation devices. C_max_ was defined as the maximum serum nicotine concentration and T_max_ was defined as the time of maximal concentration. Nicotine boost was calculated as the highest peak nicotine concentration minus the baseline nicotine concentration.

Study data were analyzed using SAS 9.3 Statistical Package. Means and frequencies were used to describe the characteristics of the sample and the outcome measures. Fisher’s Exact Test and two-sided t-tests were used as appropriate to determine differences between e-cigarette and cigarette users and between advanced and first-generation e-cigarette users. For the subjective measures, a change score was created for each measure by subtracting the pre-rating from the post-rating. A total pre and post vaping withdrawal score was created by averaging the ratings from the following measures at each time point; nervousness, difficulty concentrating, restlessness, hunger, impatience, and depression. A total pre and post vaping nicotine physical effects score was created by averaging the ratings of the following measures at each time point; nausea, dizziness, lightheaded, sweaty, headache and heart pounding. Paired t-tests were used to determine overall differences between pre and post vaping among all e-cigarette users. Independent t-tests were used to determine differences in change score for each subjective measure between advanced and first-generation e-cigarette users and between e-cigarette users overall and smokers.

## Results

### Participants and devices

**Fig 1:**
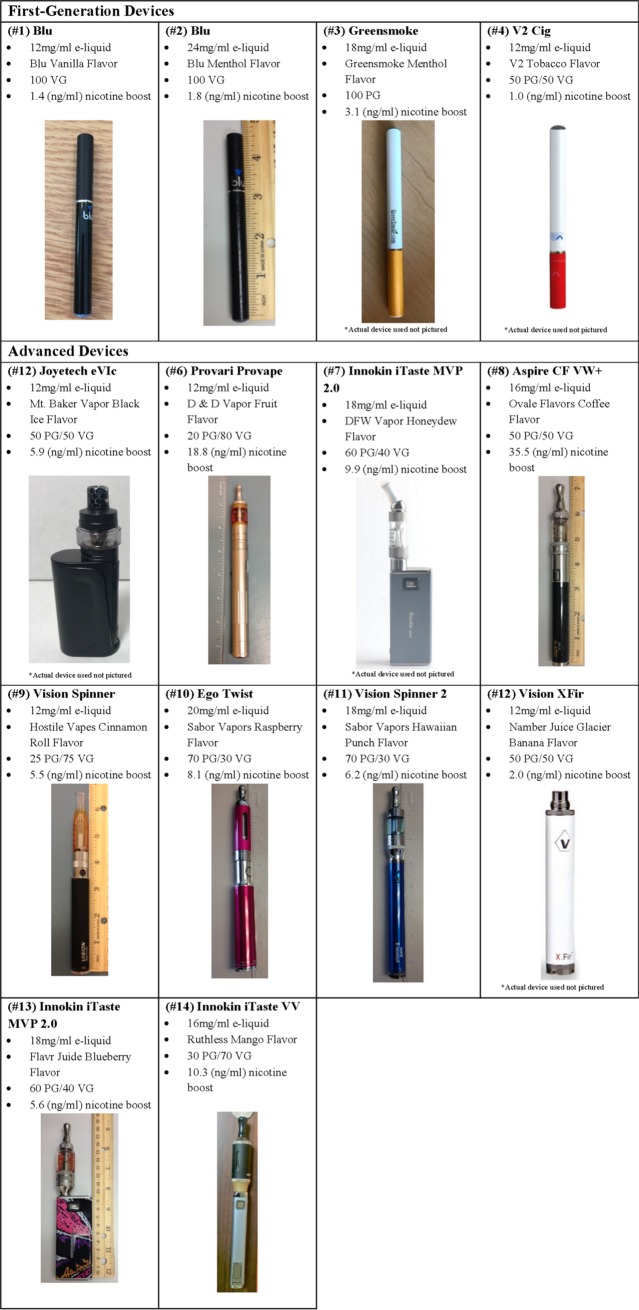
Characteristics of participants’ personal electronic cigarette (e-cigarette) devices and liquids (e-liquid). Device characteristics and pictures of the devices used by participants during the study. PG/VG = propylene glycol to vegetable glycerin ratio reported by the manufacturer.

The final sample included 14 e-cigarette users, including 10 advanced device users and 4 first-generation device users, and 10 cigarette smokers (from previously collected data) [28]. Demographic and e-cigarette related characteristics are presented in Table 1. Comparing e-cigarette users to cigarette users, cigarette smokers were significantly older and had a significantly greater exhaled CO at baseline. There were no significant differences in demographics across the e-cigarette groups. In addition, first-generation and advanced users did not differ in baseline exhaled CO, e-cigarette dependence, or concentration of nicotine used in the e-liquid. The details of each e-cigarette device used including brand, e-liquid nicotine concentration, and flavor are displayed in Fig 1.

**Table 1:** Participant characteristics by tobacco product used and by e-cigarette device group.

Characteristic	Cigarette Smokers (n = 10)	E-cigarette users (n = 14)	P-value (Cigarette vs. e-cigarette users)	First-generation Users (n = 4)	Advanced Users (n = 10)	P-value (Advanced vs. first-generation device users)
**Demographic and E-cig Characteristics**						
**Mean Age (SD)**	45.8 (11.1)	34.3 (10.8)	0.0170	34.3 (11.9)	34.3 (12.0)	0.9945
**% Male**	70.0	57.1	0.5212	75.0	50.0	0.5804
**% White**	80.0	92.9	0.3478	75.0	100.0	0.2857
**% Current Occasional Smoker**	-	21.4		25.0	20.0	0.8368
**Mean Cigarettes per day (SD)**	21.5 (3.4)	-		-	-	
**Mean Baseline Expired CO (ppm) (SD)**	6.0 (1.8)	3.1 (1.7)	0.0005	3.0 (2.9)	3.1 (1.2)	0.9512
**Mean number of months using e-cigarette (SD)**	-	9.1 (6.7)		6.5 (0.6)	10.1 (7.8)	0.1800
**Mean Penn State Electronic Cigarette Dependence Index Score (PSECDI) (SD)**	-	7.7 (3.)		8.0 (5.5)	7.2 (2.8)	0.8564
**Mean Nicotine Concentration in E-liquid (mg/ml) (SD)**	-	15.9 (3.7)		17.3 (5.1)	15.4 (3.1)	0.4185

### Electronic cigarette nicotine exposure

**Fig 2:**
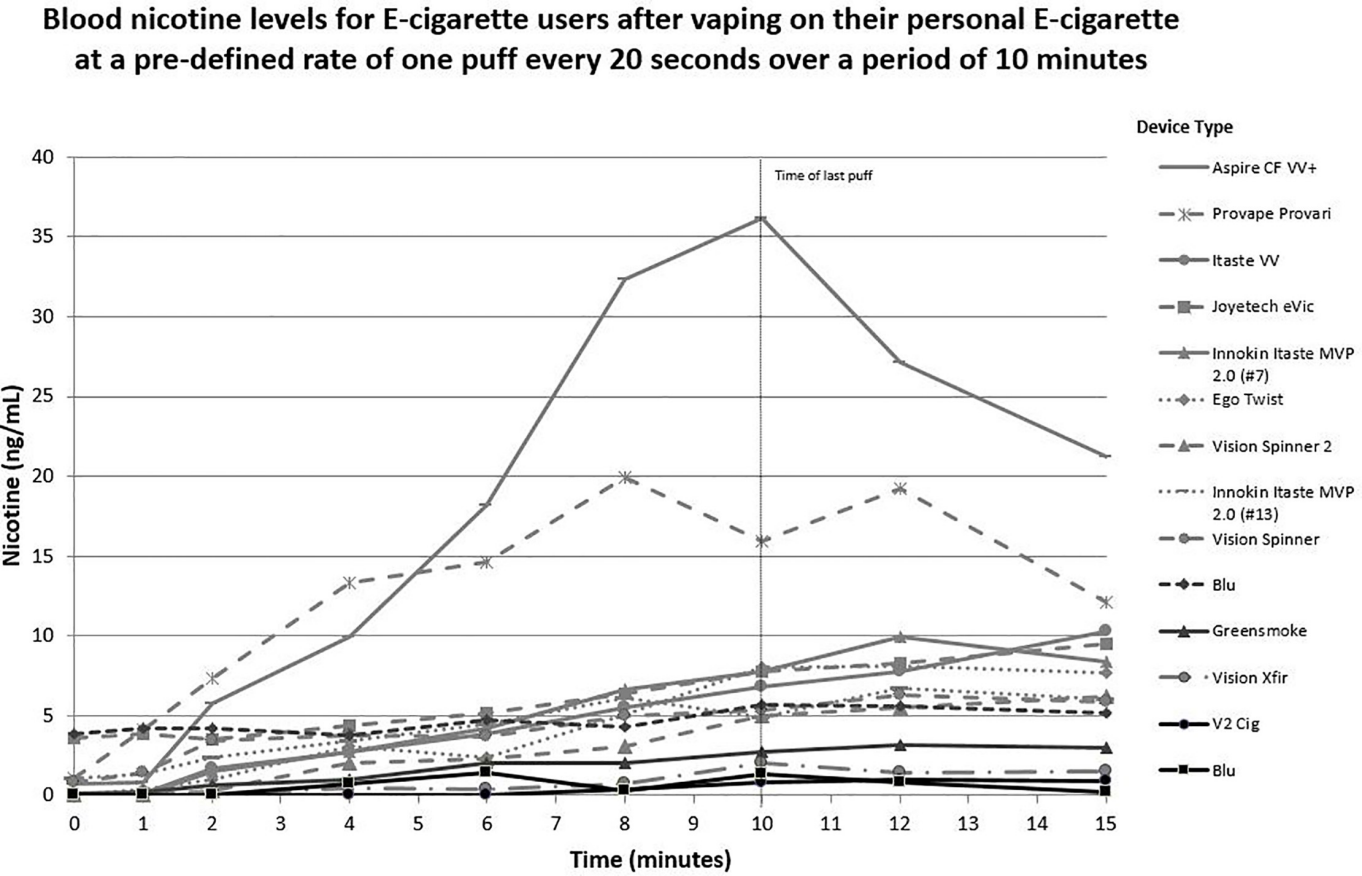
Blood serum nicotine levels for e-cigarette users after vaping on their personal e-cigarette at a pre-defined rate of one puff every 20 seconds over a period of 10 minutes. Blood serum nicotine levels for e-cigarette users after vaping on their personal e-cigarette at a standardized rate of one puff every 20 seconds for 10 minutes. Last puff on e-cigarette was taken at 10 minutes. Black lines represent first-generation devices while gray lines represent advanced devices.

**Fig 3:**
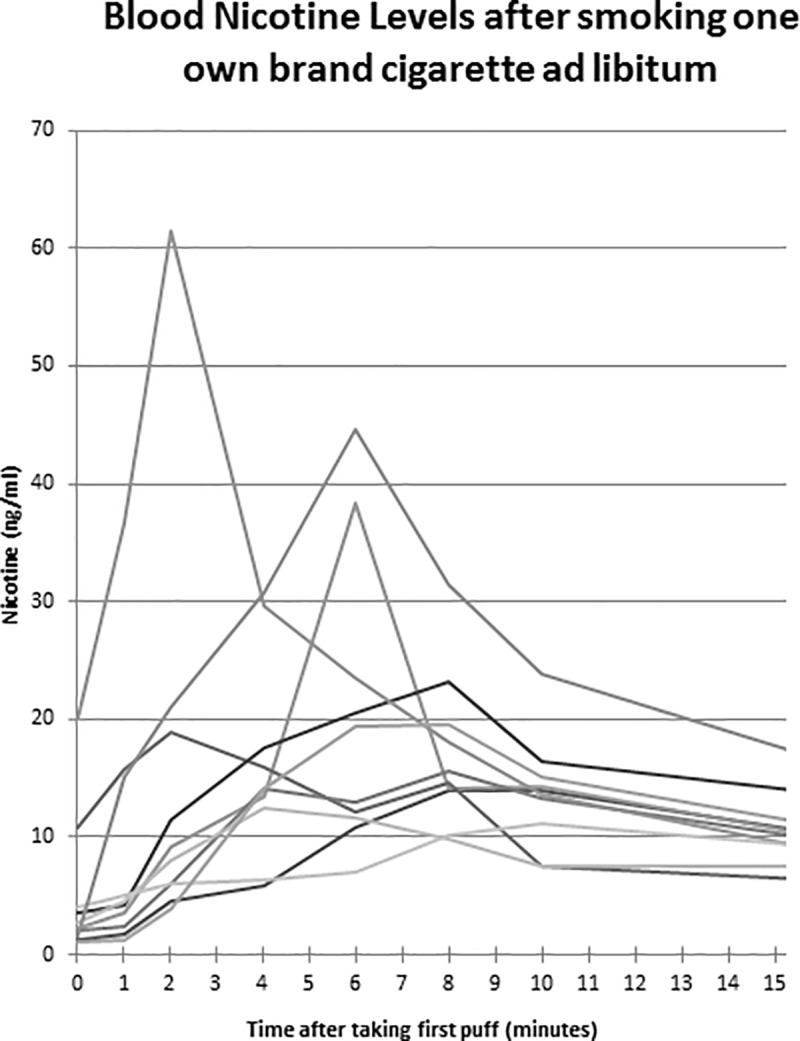
Blood serum nicotine levels after smoking one own brand cigarette ad libitum. The first 15 minutes of blood serum nicotine levels for cigarette smokers instructed to smoke their own brand of cigarette ad libitum. These data was collected in a separate comparison study (Williams et al., 2010).

**Fig 4:**
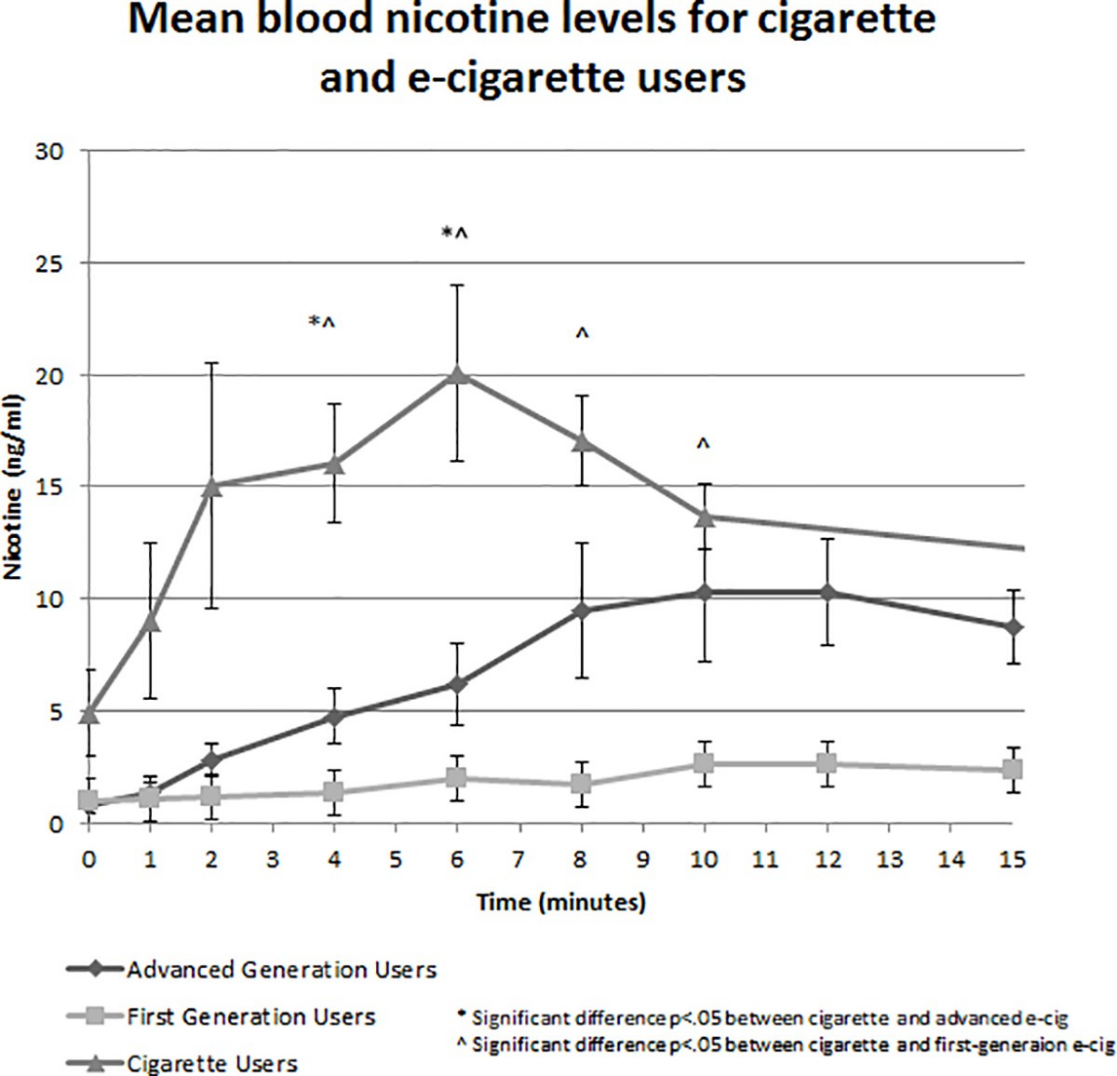
Mean blood serum nicotine levels for cigarette and e-cigarette users. Group average blood serum nicotine levels for cigarette and electronic cigarette users by device type. * denotes significant difference (p < .05) between cigarette and advanced e-cigarette users. ^ denotes significant different (p < .05) between cigarette and first-generation e-cigarette users.

Advanced devices were associated with a significantly higher max concentration (C_max_) of serum nicotine and higher nicotine boost than first-generation devices (Table 2). Fig 2 displays the blood nicotine levels for each e-cigarette participant over the course of the vaping session. Overall, advanced device users had higher increases in serum nicotine over the course of the vaping session than first-generation users. The serum nicotine levels measured during the first 15 minutes of the combustible cigarette smoking session are displayed in Fig 3. Cigarette smoking resulted in a faster and higher increase in serum nicotine than the e-cigarettes (Table 2). Fig 4 shows the average serum nicotine levels for the cigarette, advanced e-cigarette, and first-generation groups over 15 minutes of use. There was a clear differentiation of nicotine delivery by device type, with cigarettes delivering the highest levels, and first-generation devices delivering the lowest levels of nicotine. As can be seen in Fig 3, one cigarette study participant had extremely high baseline and peak levels of nicotine. Since this outlier had the potential to skew the results, the statistical comparisons of nicotine values across smokers and e-cigarette users were repeated without the inclusion of the participant. The results remained the same, with smokers showing significantly faster and higher levels of nicotine absorption than e-cigarette users.

**Table 2:** Nicotine absorption among cigarette smokers, e-cigarette users, and by e-cigarette device type.

Characteristic	Cigarette Smokers (n = 10)	E-cigarette users (n = 14)	P-value (Cigarette vs. e-cigarette users)	First-generation Users (n = 4)	Advanced Users (n = 10)	P-value (Advanced vs. first-generation device users)
**Mean Baseline Nicotine Level (ng/ml) (SD)**	4.9 (6.0)	.81 (1.3)	0.0597	1.0 (1.9)	0.7 (1.1)	0.7359
**Mean time smoking cigarette from first to last puff (min) (SD)**	5.2 (1.1)	-		-	-	
**Mean Tmax (min) (SD)**	6.2 (2.9)	11.5 (2.6)	0.0001	10.0 (2.8)	12.1 (2.4)	0.1808
**Mean Cmax (ng/ml) (SD)**	25.9 (16.7)	9.0 (9.2)	0.0043	2.8 (2.1)	11.5 (9.8)	0.0231
**Mean Nicotine Boost (ng/ml) (SD)**	21.0 (13.9)	8.2 (9.2)	0.0128	1.8 (0.9)	10.8 (9.8)	0.0177

### E-cigarette subjective effects

On average, e-cigarette users experienced significant reductions in self-reported withdrawal (Table 3). While there was an overall reduction in withdrawal after the vaping session, there were no significant differences in withdrawal symptom reduction between the advanced and first-generation users after the vaping sessions. E-cigarette use was not associated with a significant increase in nicotine side effects and there was no difference between groups. Finally, craving was significantly reduced overall after e-cigarette use and there was no significant difference in craving relief between first-generation and advanced device users.

**Table 3:** Subjective ratings prior to and after e-cigarette use overall and by e-cigarette device group.

Measure Mean Score (SD)	All E-cigarette users			E-cigarette users by device group		
	Pre-Vape Score (n = 14)	Post-Vape Mean Score (n = 14)	P-value (Pre vs. Post Vaping)	First-generation Mean Change Score (Post minus Pre) (n = 4)	Advanced Mean Change Score (Post minus Pre) (n = 10)	P-value (First-generation vs. Advanced group)
**Withdrawal Score**	19.01 (17.95)	10.00 (8.40)	0.0319	-11.36 (18.27)	-8.07 (13.02)	0.7086
** Nervous**	19.29 (24.88)	3.64 (7.28)	0.0353	-10.50 (26.60)	-17.70 (25.38)	0.6442
** Anxious**	18.79 (28.95)	4.57 (9.87)	0.0368	-24.50 (31.88)	-10.10 (18.49)	0.3053
** Difficulty Concentrating**	21.00 (29.83)	5.93 (13.66)	0.0150	-27.25 (21.28)	-10.20 (18.49)	0.1597
** Restlessness**	17.71 (27.99)	6.00 (13.80)	0.0742	-31.75 (35.04)	-3.70 (8.80)	0.2076
** Hunger**	35.21 (25.72)	43.07 (31.54)	0.2171	25.75 (28.24)	0.70 (16.64)	0.0577
** Impatient**	16.14 (23.99)	5.57 (11.83)	0.0438	-10.75 (19.55)	-10.50 (18.05)	0.9821
** Depression**	4.93 (10.94)	1.21 (4.54)	0.1950	-0.50 (1.00)	-5.00 (11.94)	0.2669
**Nicotine Side Effects**	8.04 (10.36)	10.64 (11.08)	0.5390	2.79 (25.47)	2.53 (11.36)	0.9788
** Nauseous**	5.71 (14.53)	7.71 (16.33)	0.7349	-10.00 (20.00)	6.80 (21.28)	0.2006
** Dizzy**	7.71 (10.09)	16.71 (21.26)	0.1344	19.50 (35.63)	4.80 (12.28)	0.4744
** Lightheaded**	14.57 (19.22)	22.86 (23.37)	0.3310	19.50 (55.02)	3.80 (16.54)	0.6117
** Sweaty**	2.93 (6.45)	6.79 (21.25)	0.5377	-5.00 (10.00)	7.40 (25.86)	0.3789
** Headache**	11.71 (20.76)	8.57 (10.03)	0.5577	5.25 (28.30)	-6.50 (15.51)	0.3288
** Heart Pounding**	5.57 (13.40)	1.21 (4.00)	0.2481	-12.50 (25.00)	-1.10 (3.60)	0.4297
**Salivation**	7.50 (17.51)	1.14 (3.74)	0.2049	-20.00 (30.82)	-0.90 (5.13)	0.3038
**Constipation**	5.86 (11.31)	2.57 (6.55)	0.1440	0.00 (0.00)	-4.60 (9.14)	0.1461
**Craving**	57.43 (32.46)	27.21 (31.36)	0.0126	-38.00 (50.38)	-27.10 (36.41)	0.6562
**Drowsiness**	31.29 (36.90)	19.71 (25.34)	0.1466	9.25 (35.70)	-19.90 (21.01)	0.0771
**Craving for sweets**	18.79 (29.64)	13.57 (24.03)	0.3937	-1.25 (2.50)	-6.80 (26.36)	0.5260

## Discussion

This study evaluated the nicotine delivery of e-cigarettes and compared the nicotine delivery of two classes of e-cigarettes, first-generation and advanced devices, to the nicotine delivery of cigarettes. We found that advanced e-cigarette devices were associated with a greater maximum concentration of nicotine and a greater nicotine boost compared with first-generation devices. These results support prior research showing that first-generation devices deliver less nicotine than advanced devices [13, 22]. Even among the current sample of experienced users, vaping with their personal devices, the nicotine delivery of advanced devices exceeds that of first-generation devices. This may help to explain users’ preferences for second-generation advanced devices over first-generation devices [5, 22, 29]. In addition, while many studies of nicotine absorption measure baseline blood nicotine and then again immediately after a period of vaping (e.g. 5 minutes)[10, 18, 19], the present study was able to assess nicotine absorption during vaping and more accurately assess the Tmax by taking 5 blood samples while the participants were actively vaping (1, 2, 4, 6, and 8 minutes after starting vaping, as well as immediately after they finished vaping, at minutes 10, 12 and 15. We found that the average Tmax for e-cigarette users overall was at 11.5 minutes, with no differences between the users of first-generation and advanced users.

We also found that despite using similar devices with an e-liquid nicotine concentration greater than 12mg/ml, there was noticeable variability in nicotine delivery within both the first-generation and advanced user groups. As discussed in recent studies, the amount and rate of nicotine delivered may depend on the user's technique, such as the length of the puff, or the characteristics of the device, such as nicotine concentration or flavor [14, 18, 20, 30]. A recent study by St. Helen et al, reported that pH levels in e-liquids vary by flavor, which may impact the rate of nicotine absorption depending on the flavor used [30].

Overall, e-cigarette users experienced a decrease in withdrawal and craving after use while experiencing no side effects related to ingestion of nicotine. While there were overall reductions in withdrawal and craving after vaping, there were no differences in withdrawal or craving reduction between users of first-generation and advanced devices. Similarly, a previous study found that first-generation and advanced devices were equally effective in reducing the urge to smoke and withdrawal symptoms, however this study also found that advanced devices were perceived as more satisfying [22]. Since first-generation users receive significantly less nicotine but experience a similar reduction in withdrawal and craving compared to advanced users, this suggests that there may be something about the behavior of vaping that helps to reduce withdrawal and craving. Future research would benefit from assessing other sensory factors that may create satisfaction with use other than nicotine [31].

When compared to cigarettes, e-cigarettes were associated with a lower boost in blood nicotine levels, however some e-cigarette devices did deliver nicotine similarly to a cigarette. One e-cigarette user was able to obtain a nicotine boost of 35.5ng/ml, which is higher than the mean nicotine boost obtained by cigarette smokers. Our findings support those of previous studies that suggest some advanced devices are able to deliver blood nicotine concentrations similar to that obtained from cigarettes [13, 17–19, 32]. In addition, while users of e-cigarettes had a longer time to maximal concentration, this may be due to the different puffing protocols for the smokers and e-cigarette users. For both e-cigarette users and cigarette smokers, the time of maximal concentration came approximately 1.2–1.5 minutes after the last puff.

Given that there were only four first-generation users in the study, statistical comparisons to advanced users should be interpreted with caution and are underpowered to identify small effects. Our difficulty in recruiting participants who regularly used first-generation devices was not surprising given that first-generations deliver small amounts of nicotine in comparison to advanced devices [13] and because long-term users are likely to transition to advanced devices [5]. In addition, this study reports on data collected from 2014–2015. While the variety and types of e-cigarette devices available have increased since this study was conducted, most of the e-cigarettes used in the study remain on the market and the findings from this study are also important to understanding data on e-cigarette use during that time period. For example, many studies published today utilize national data that was collected during 2014–2015, when most users were utilizing devices very similar to the ones measured in this study [33–37]. Additionally, this study utilized visual analog scales to have participant’s rate withdrawal symptoms and side effects of e-cigarette use on a scale of 0–100. This study used items that are also included in the Minnesota Nicotine Withdrawal Scale (MNSW) [25], a commonly accepted measure of withdrawal that asks participants to rate their withdrawal over the past 24 hours. We utilized items that asked participants to rate how they were feeling right now, and the VAS enabled participants to record their ratings rapidly by simple clicking along a continuum. This scoring method has not been as thoroughly validated as the traditional 0–4 rating method used in the MNWS.

Finally, this study included participants who used a nicotine concentration of at least 12mg/ml and asked participants to vape on an intensive puffing schedule. While it is now known that e-cigarette users can absorb a sizeable amount of nicotine using lower nicotine concentrations and a less intensive puffing schedule, when this study was designed, it was hypothesized that e-cigarettes did not deliver very high doses of nicotine. We chose to include only those with a higher nicotine concentration and to use an intensive puffing schedule to ensure that participants absorbed some nicotine during use. Despite this intensive puffing schedule, first-generation users absorbed only a very small amount of nicotine (boost of 2.8 ng/ml).

An important implication of our study results is that the first-generation e-cigarettes many smokers first try [5] only deliver small amounts of nicotine, even after very intensive puffing (30 puffs over 10 minutes). It is therefore not surprising that these types of devices do not result in impressive rates of smoking cessation in randomized trials [38] or in cohort studies [39]. The finding that most e-cigarettes deliver significantly lower blood nicotine concentrations is consistent with the evidence that e-cigarette users rate themselves as less addicted to their product than cigarette smokers [23, 40, 41]. Future evaluations of the role of e-cigarettes in smoking cessation should pay greater attention to the actual nicotine delivery of the device under examination, as this clearly varies considerably.

In conclusion, this study found that advanced e-cigarette devices delivered significantly more nicotine than first-generation devices but significantly less than combustible cigarettes. Despite obtaining less nicotine, users of first-generation devices experienced the same reductions in withdrawal and craving as advanced device users. These findings provide a basis for understanding dependence among e-cigarette and cigarette users and could have implications for future e-cigarette product standards.
